# Benchmarking an 11-qubit quantum computer

**DOI:** 10.1038/s41467-019-13534-2

**Published:** 2019-11-29

**Authors:** K. Wright, K. M. Beck, S. Debnath, J. M. Amini, Y. Nam, N. Grzesiak, J.-S. Chen, N. C. Pisenti, M. Chmielewski, C. Collins, K. M. Hudek, J. Mizrahi, J. D. Wong-Campos, S. Allen, J. Apisdorf, P. Solomon, M. Williams, A. M. Ducore, A. Blinov, S. M. Kreikemeier, V. Chaplin, M. Keesan, C. Monroe, J. Kim

**Affiliations:** 1 IonQ, Inc., College Park, MD 20740 USA; 2 Joint Quantum Institute and Department of Physics, University of Maryland, College Park, MD 20742 USA; 3 Department of Electrical and Computer Engineering, Duke University, Durham, NC 27708 USA

**Keywords:** Atomic and molecular physics, Quantum information

## Abstract

The field of quantum computing has grown from concept to demonstration devices over the past 20 years. Universal quantum computing offers efficiency in approaching problems of scientific and commercial interest, such as factoring large numbers, searching databases, simulating intractable models from quantum physics, and optimizing complex cost functions. Here, we present an 11-qubit fully-connected, programmable quantum computer in a trapped ion system composed of 13 ^171^Yb^+^ ions. We demonstrate average single-qubit gate fidelities of 99.5$\%$, average two-qubit-gate fidelities of 97.5$\%$, and SPAM errors of 0.7$\%$. To illustrate the capabilities of this universal platform and provide a basis for comparison with similarly-sized devices, we compile the Bernstein-Vazirani and Hidden Shift algorithms into our native gates and execute them on the hardware with average success rates of 78$\%$ and 35$\%$, respectively. These algorithms serve as excellent benchmarks for any type of quantum hardware, and show that our system outperforms all other currently available hardware.

## Abstract (web summary)

The growing complexity of quantum computing devices makes presents challenges for benchmarking their performance as previous, exhaustive approaches become infeasible. Here the authors characterise the quality of their 11-qubit device by successfully computing two quantum algorithms.

## Introduction

Small universal quantum computers that can execute textbook quantum circuits exist in both academic^1–5^ and industrial^6–10^ settings. With a range of 2–72 qubits and sufficient fidelity for only tens of entangling gates, these devices and the underlying qubit implementations can be difficult to compare. Even within the trapped ion platform, there is large diversity in atomic species, system architectures, and gate implementations. Trapped ion systems with one to two qubits have shown single-qubit gate fidelities of 99.9999%^11^ with microwave-based operations and better than 99.99% fidelity with laser-based operations^12,13^, state preparation and measurement (SPAM) error below $1{0}^{-4}$^11,14^, and two-qubit gates with fidelities exceeding 99.9%^12,13^. Algorithms have been executed on up to seven trapped-ion qubits^15^ and, while not optimized for universal quantum computing, quantum simulators with more than 50 ions have modeled fundamental quantum systems including Ising chains^16^ and quantum magnetism^17^.

Benchmarking across implementations needs to be both universal across platforms and agnostic to the differences in the underlying hardware. In traditional computing, the performance of computers is measured by executing a set of benchmark problems representing various use-case scenarios, to provide users with an estimate of how the computers would perform in their specific applications. Canonical quantum algorithms demonstrate unambiguous advantage of quantum computers over classical computation, and provide verifiable outcomes to assess successful execution of the algorithm. Therefore, they can serve as ideal candidate problems for benchmarking the performance of any quantum computers. These benchmark algorithms exercise the full hardware/software stack. A hardware-specific compiler breaks down algorithms into the target hardware’s native gate set, optimizing for qubit connectivity, gate times, and coherence^18^ to enhance the system’s performance. After execution on the hardware, the measurements can be directly compared with the expected output state to determine the accuracy of the device. This accuracy can then be compared with other devices that have compiled and run the same algorithm^19^.

**Fig. 1:**
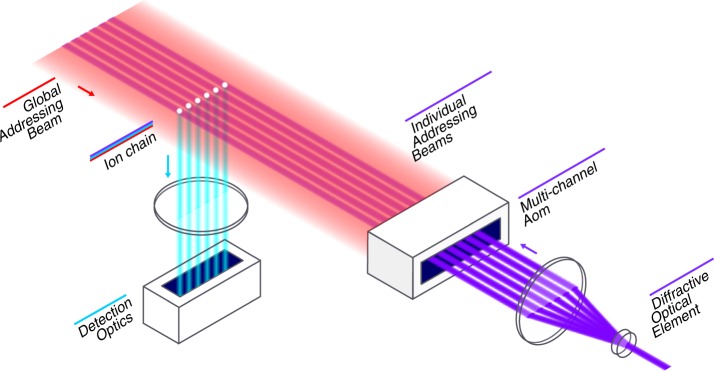
Schematic of the hardware. A linear chain of ions is trapped near a surface electrode trap (trap is not shown). Lasers at 369 and 935 nm (not shown) illuminate all of the ions during cooling, initialization, and detection. Each ion’s fluorescence is imaged through a 0.6 numeric aperture lens (detection optics) and directed onto individual photomultiplier tube channels. Two linearly polarized counterpropagating 355 nm Raman beams are aligned to each qubit-ion, a globally addressing beam that couples to all of the qubits (red) and an individual addressing beam that is focused onto each ion (blue). Acousto-optic modulators (AOMs) modulate the frequency and amplitude of each of these beams to generate single-qubit rotations and XX-gates between arbitrary pairs of qubit ions.

We benchmark two algorithms on an IonQ-trapped ion quantum computer, shown schematically in Fig. 1. Our qubit register is comprised of a chain of trapped ^171^Yb^+^ ions, spatially confined near a microfabricated surface electrode trap^20^, separating this work from similar implementations in more macroscopic traps^3,18^. By using a microfabricated trap, the underlying hardware of this quantum computer is more extensible than a traditional macroscopic trap. This is due in large part to the highly reproducible nature of microfabricated devices. In addition to this advantage, microfabricated surface traps have many more control electrodes, which allows for the fine control of the trapping potential. This becomes practically very important when trying to maintain equal spacing confinement in long chains of ions. To the best of our knowledge the largest similar algorithm implementation using a surface electrode trap was limited to three qubits^21^; for this work, we loaded 13 ions, the middle 11 of which were used as qubits. The two end ions allowed for a more uniform spacing of the central 11 ions. However, on this same apparatus we have successfully loaded over 150 ions and have done selective single qubit rotations on subsets of chains of up to 79 qubits. The choice to use 11 qubits was informed by the number of gates required for full oracle implementations, our underlying gate fidelities, and the time required to run all of the oracles.

## Results

### Gate implementation and characterization

The ions are laser-cooled close to their motional ground state using a combination of Doppler and resolved sideband cooling. We encode quantum information into the hyperfine sublevels, $$\left|0\right\rangle \equiv \left|F=0,{m}_{F}=0\right\rangle$$ and $$\left|1\right\rangle \equiv \left|F=1,{m}_{F}=0\right\rangle$$ of the $${}^{2}{S}_{1/2}$$ ground state. At the beginning of each computation, each qubit is initialized to $$\left|0\right\rangle$$ via optical pumping with high accuracy. After qubit operations (described below), we read out the state of all of the qubits simultaneously by directing laser light resonant with the $${}^{2}{S}_{1/2}$$$$\left|F=1\right\rangle$$ to $${}^{2}{P}_{1/2}$$ transition, imaging each ion onto an independent detector and thresholding the photon counts to determine if each qubit was in the $$\left|1\right\rangle$$ (spin up) or $$\left|0\right\rangle$$ (spin down) state. Thresholding is done by taking a histogram of the collected photons and discriminating between collecting on average zero photons for the $$\left|0\right\rangle$$ state and 10 photons on average for the $$\left|1\right\rangle$$ state. Thresholding is a sufficient discriminating function in our system because our detectors are highly isolated from one another resulting in detection crosstalk between adjacent ions below a part in 10^4^.

A two-photon Raman transition drives single-qubit and two-qubit coherent operations by applying a pair of counter-propagating beams from a mode-locked pulsed 355 nm laser^22^. One of these beams globally addresses all of the ions simultaneously, while the other beam addresses any of the ions individually (Fig. 1). The individually addressing beams pass through a multi-channel acousto-optic modulator (AOM), which allows for the simultaneous modulation of the phase, frequency, and amplitude of each beam. To perform a single-qubit gate, we tune the frequency difference between Raman beams to resonantly drive a spin–flip transition ($\left|1\right\rangle \leftrightarrow \left|0\right\rangle$). In order to perform a two-qubit gate, we off-resonantly drive motional sideband transitions to generate an XX-interaction^23^. Both the global and individual beams are directed over the trap surface perpendicular to the axis of the ion chain to excite one principal axis of motion transverse to the chain axis. Individual addressing allows us to perform single-qubit and two-qubit gates on any targeted qubits.

**Fig. 2:**
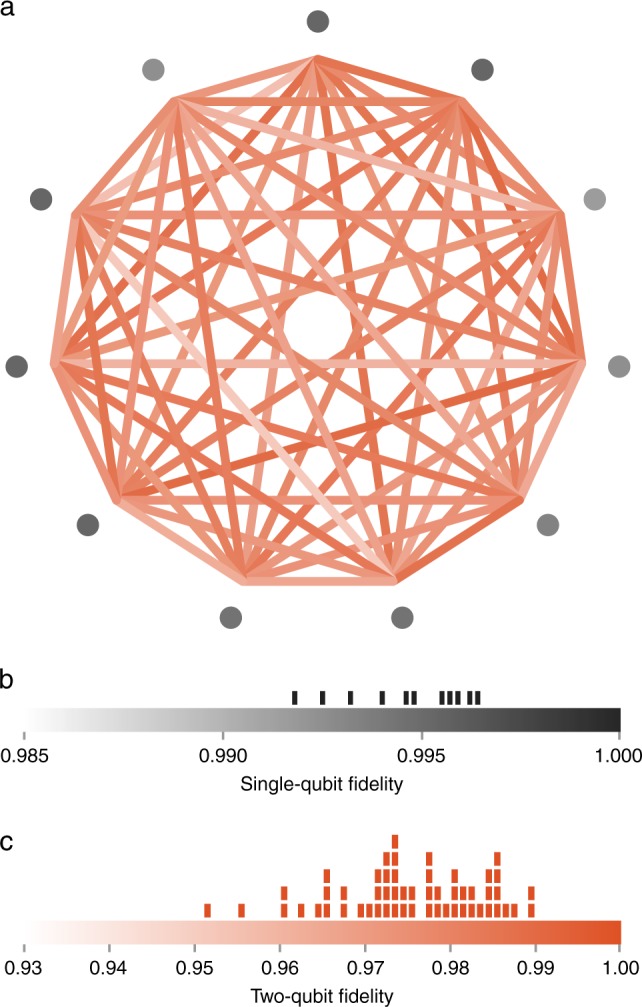
Fidelity of native gates. For each qubit pair, we perform an XX-gate and measure the joint populations of the qubit pair as a function of an analysis pulse phase angle. The fidelity of two-qubit gates are plotted as a color scale in the illustration of our all-to-all connectivity in **a**. For each qubit, we perform randomized benchmarking to determine the fidelity of the single-qubit gates shown in **b**, which are plotted as the color scale of the nodes in **a**. We use maximum-likelihood estimation to extract fidelities from the parity and joint-population measurement shown in **c**. The average two-qubit raw fidelity is 97.5% and all two-qubit gates perform in the range [95.1%, 98.9%]. The distribution of these fidelities are depicted in the histograms above the color bars shown in **b**, **c**. The fidelity of all single-qubit gates are enumerated in Supplementary Table 1 and all two-qubit pairs are enumerated in Supplementary Table 2 of the extended data.

Native two-qubit entangling XX-gates are achieved by driving a spin-dependent force^23^. Using an amplitude-modulated (AM) pulse on any selected pair of qubits, we address multiple transverse motional modes of the ion chain to mediate a spin–spin Ising interaction between qubits^24^. To achieve high fidelity, the amplitude modulation is calculated to simultaneously decouple all motional modes from the spin at the end of the gate operation. Additionally, these pulse shapes are designed to provide robustness against frequency drift of motional modes and suppress residual off-resonant carrier excitation during the XX-gate^24–27^. This gate, in conjunction with single-qubit rotations, forms a universal gate set for performing circuit model quantum computation. Since the XX-gates are mediated by the collective motion of the ion chain, we have all-to-all connectivity between qubits, allowing two-qubit gates to be executed between any qubit pair (Fig. 2a).

We perform randomized benchmarking^28^ to characterize the single-qubit operations on each ion of the 11-qubit chain. We apply a randomly chosen sequence of $\pi /2$ gates with length $L$ about the $X$ and $Y$ axes. In between each of these $\pi /2$ gates, we either add a $\pi$ rotation about the $X$, $Y$, or $Z$-axis, or an identity operation (leaving the qubit idle for the duration of a gate). A final $\pi /2$ gate is chosen such that the final state is in the $Z$ computational basis (i.e. $\left|0\right\rangle$ or $\left|1\right\rangle$). We measure the overlap between the measured and expected output states across 500 iterations for at least 24 sequences for each $L\in \{2,4,6,8,10,12\}$. The fidelity of our single-qubit $\pi /2$ gate is then determined by fitting the resulting overlap as a function of sequence length to a power law, $B{p}^{L}+\frac{1}{2}$. Here, the base $p$ is the gate fidelity and the intercept $B+\frac{1}{2}$ is the SPAM fidelity, equivalent to measuring the ion after a single $\pi$ rotation when it is in either state $\left|0\right\rangle$ or $\left|1\right\rangle$. For a chain of 11 qubits, we measure an average single-qubit fidelity of 99.5% (Fig. 2b) and an average SPAM fidelity of 99.3%.

To quantify the performance of our two-qubit gates and estimate their fidelity, we measure the state fidelity of the Bell state $\frac{1}{\sqrt{2}}(\left|00\right\rangle +{{\mathrm{{e}}}}^{i\phi }\left|11\right\rangle )$ prepared using a single XX-gate by performing partial tomography of the state^12,13^. The diagonal terms of the two-qubit density matrix are extracted by measuring the populations in the even parity states. The populations are measured when the overall AM pulse height for the XX-gate is tuned to achieve maximal entanglement such that the even-parity two-qubit states, ${P}_{00}$ and ${P}_{11}$, are equal (${P}_{00}={P}_{11}$). The off-diagonal elements are obtained from the amplitude $\Phi$ of a parity oscillation, where the parity is given by ${P}_{00}+{P}_{11}-{P}_{01}-{P}_{10}$ (${P}_{01}$ and ${P}_{10}$ are the populations of the odd parity two-qubit states). The fidelity can then be calculated as $F=\frac{1}{2}$(${P}_{00}+{P}_{11}+\Phi$)^13^. We use maximum-likelihood estimation on experimentally observed data to extract the parameters of the fidelity expression^13^. We have performed this analysis for all 55 pairs of qubits in the 11-qubit chain (Fig. 2c) and measure an average fidelity of 97.5% with a minimum and maximum fidelity of 95.1${}_{-0.7}^{+0.5} \%$ and 98.9${}_{-0.3}^{+0.1} \%$, respectively. The uncertainty here is determined by a statistical confidence interval on the maximum-likelihood estimation. The reported fidelity represents a lower bound of the Bell state creation as we do not correct for SPAM errors on the two-qubit states or errors in single-qubit rotations used to observe the parity oscillations of the Bell state, which on average are 0.7$\%$ and 0.5$\%$ respectively.

### Bernstein–Vazirani (BV)

To benchmark our system, we implement two well-known algorithms: the BV and Hidden Shift (HS). Both of these algorithms have previously been run on trapped-ion^3,18,21^ and superconducting^4,18,19^ systems of up to five qubits. By comparing the results of this algorithm to the ideal result, we obtain a direct measure of the system performance, which accounts for our native gates, connectivity, coherence times, gate duration, and all other isolated metrics of system performance. These results can be used as part of a suite of algorithms to compare our hardware with other systems. The qubit number in these results is higher than any comparable published BV or HS results using a programmable quantum computer^3,4,18,19,21^.

**Fig. 3:**
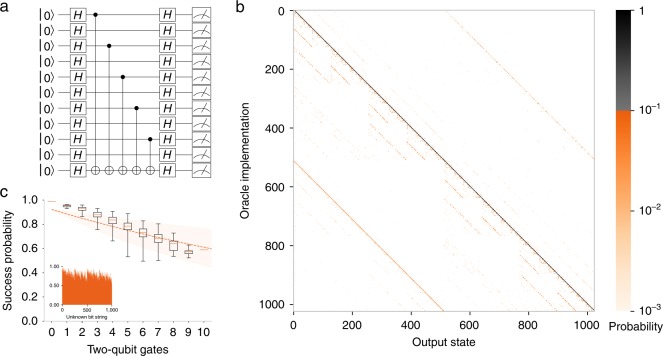
Bernstein–Vazirani (BV) algorithm. **a** Shows a textbook implementation of the BV algorithm with hidden bit string 1010101010. **b** Shows the full output distribution for all 1024 oracle implementations calculated from 500 iterations of each oracle after conditioning on the ancilla. **c** Shows the probability (inset plot) of detecting the encoded hidden bit string for all 1024 oracle implementations, as a function of the number of ones in the binary representation of the unknown bit string, which is equivalent to the number of two-qubit gates (*n*), which is maximally 10 in the case of this algorithm. The boxplots highlight the minimum, first quartile, median, third quartile, and maximum of the data. Note that there is only one oracle implementation for *n* = 0, 10, which explains the lower observed variances for these points. In contrast, there are many more oracles that consist of five two-qubit gates, where each included gate has slightly different fidelity. This leads to increased variance across the full set of five two-qubit gate oracle implementations. The shaded area spans the expected fidelity (excluding crosstalk errors) ${{\mathcal{F}}}_{\,\text{2Q}}^{\text{n}\,}{{\mathcal{F}}}_{\,\text{1Q}\,}^{2(\,\text{n}\,+1)}{{\mathcal{F}}}_{\,\text{SPAM}\,}^{10}$ (where ${{\mathcal{F}}}_{\text{2Q}}$ is the fidelity of two-qubit gates, ${{\mathcal{F}}}_{\text{1Q}}$ is the fidelity of single-qubit gates, and ${{\mathcal{F}}}_{\text{SPAM}}$ is the average SPAM fidelity) if all of our gates share the best measured fidelity or, alternatively, all share the worst fidelity. The result of a shared average fidelity is plotted as a dashed line. The average probability of success is 78$\%$ with 899 out of the 1024 oracle implementations exceeding the $2/3$ BQP single-shot success threshold.

The BV algorithm is an oracle problem in which the user tries to determine an unknown bit string $c$ of size $N$, implemented by a specific oracle. The algorithm takes a binary input string $x$ and performs a controlled inversion of an ancillary bit or qubit based on the bit-wise product of the input and the unknown bit string $c$ modulo two, $f(x)=c\cdot x \, ({\mathrm{mod}} {\,} 2)$^29^. For a quantum BV implementation (example shown in Fig. 3a), a single quantum query is sufficient to determine the bit string $c$^30^. This is a linear improvement over the best classical algorithm, which requires $N$ queries. The BV algorithm was developed to help separate a class of problems that can be solved in polynomial time on a quantum computer with bounded errors, bounded-error quantum polynomial (BQP), from its classical counterpart. For an algorithm to belong to BQP, it must succeed with probability at least 2/3 on all possible inputs after only a polynomial number of repetitions. This implies that the single-shot success probability must exceed 1/2 for all inputs, which allows reaching the 2/3 threshold by classical majority vote on multiple repetitions. This way, the 2/3 threshold success for the algorithm to be above the BQP theshold may be met with a polynomial number of queries^29^.

We compile the BV algorithm into our native gate set, comprised of single-qubit rotations and two-qubit XX-gates. Optimization during compilation reduces the number of needed gates compared to naively translating the textbook circuit from CNOT gates into rotations and XX-gates. The compilation exploits the full connectivity of our qubits, since we do not need SWAP operations. The implementation of BV requires a single-qubit ancilla and a register of $N$ qubits. There are ${2}^{N}$ possible bit strings, therefore for our 10-qubit register there are 1024 possible oracle implementations. We measured each implementation 500 times, conditioned upon on the measured ancilla state, and plot the output distribution in Fig. 3b. Each oracle implementation has, depending on the unknown bit string $c$, between 0 and 10 two-qubit gates between the ancilla and the qubit register, corresponding to the number of ones in the binary representation of the unknown bit string. The process matrix that maps the encoded oracle to the measured output state is nearly diagonal, resulting in a highly peaked distribution at the encoded oracle. For our system, the average overlap between output state and unknown bit string is 78$\%$ (Fig. 3c), where 87.8$\%$ of oracle implementations achieve the 2/3 success criteria defined by BQP. Conditioning the output on the ancilla state results in a 5.1 percentage point increase in the raw success probability of 73–78$\%$ and an 14.5 percentage point increase in the fraction of oracle implementations above the BQP threshold from 73.3$\%$ to 87.8$\%$. The average overlap in Fig. 3c decreases with the number of two-qubit gates needed in the oracle. The off-diagonal components of the process matrix show errors since these should all have zero population. In Fig. 3b, the dominant error is single-qubit bit-flips from $\left|1\right\rangle$ to $\left|0\right\rangle$ during the measurement process, which appear as faint diagonals in the lower left quadrant of the figure. However, even for the oracle implementation where we have the lowest success probability, the next-most-probable state is still four times less likely than the correct string.

### Hidden shift

**Fig. 4:**
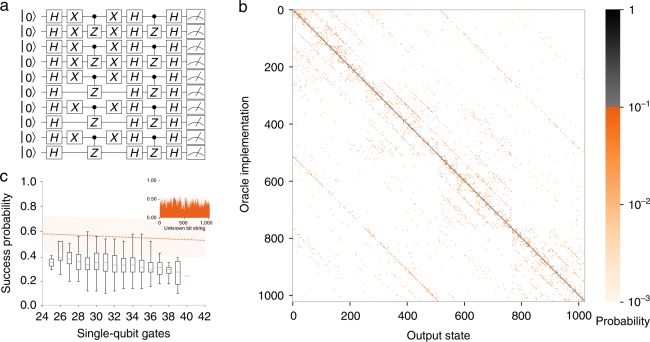
Hidden Shift (HS) algorithm implementation on 10 qubits. **a** Shows a textbook implementation of the HS algorithm with hidden shift 1111101010. The circuit for each oracle was measured at least 50 times. We trace out the spectator ion and interpret the binary output state of the 10-qubit register as an integer. The full output distribution is shown in **b**. **c** Shows the probability of detecting the encoded shift *s* for each of the 1024 oracle implementations versus the number of single-qubit gates (*m*). The shaded area represents the expected fidelity ${{\mathcal{F}}}_{\,\text{2Q}\,}^{10}{{\mathcal{F}}}_{\,\text{1Q}}^{\text{m}\,}{{\mathcal{F}}}_{\,\text{SPAM}\,}^{10}$ (where ${{\mathcal{F}}}_{\text{2Q}}$ is the fidelity of two-qubit gates, ${{\mathcal{F}}}_{\text{1Q}}$ is the fidelity of single-qubit gates, and ${{\mathcal{F}}}_{\text{SPAM}}$ is the average SPAM fidelity) if all of our gates share the best measured fidelity or, alternatively, all share the worst fidelity. Additionally, the success probability is reduced by crosstalk onto adjacent ions from the individually addressing Raman beams. This error impacts the result of the HS oracles more than the BV oracles. The result of a shared average fidelity is plotted as a dashed line. The average probability of success is 35$\%$, and 1017 of the 1024 oracle implementations correctly return the hidden shift as the maximal probability state.

The HS algorithm consists of two N-bit to N-bit function oracles *f* and *g*, which are the same up to a shift by a hidden bit string $s$, such that $g(x)=f(x+s)$. The goal is to determine the HS $s$ by querying the oracles. In our implementation^31^ of the HS algorithm, the oracles are inner product or bent functions $f={\sum }_{i}{x}_{2i-1}{x}_{2i}$ and $g=f(x+s)$, where $x$ is the input and ${x}_{i}$ is the $i$-th bit of $x$ (an example is shown in (Fig. 4a). Classically it can be shown that determining the shift $s$ requires $\sqrt{{2}^{N}}$ queries where $N$ is the length of the bit string $s$. On a quantum computer, in principle, the shift can be read out in a single query^31,32^. In contrast to the BV algorithm, the quantum implementation of the HS algorithm shows an exponential reduction in the number of queries to the oracle compared to a classical computer^31^.

As with the BV algorithm, we compile the HS algorithm into our native gates. There are ${2}^{N}$ available oracle implementations corresponding to the ${2}^{N}$ possible hidden bit strings $s$. We execute all 1024 possible implementations on our 10-qubit register (Fig. 4b). The correct output state is the state corresponding to the HS. The average overlap between the output state and $s$ was 35$\%$ (Fig. 4c), and of the 1024 oracles, 1017 had most likely output states corresponding to the shift. The success probability for HS is lower and more uniform than that of BV because all of the oracles have the same number of two-qubit gates (10) and many more single-qubit gates (25–40). Every oracle implementation in HS has at least as many gates as the most challenging BV oracle implementation and therefore is more difficult. Given our average single-qubit and two-qubit fidelities, we would not expect to surpass the BQP threshold for the HS oracles. However, the successful determination of the shift was achieved much more frequently than if we sampled a classical distribution where the success probability would have been 0.1%.

## Discussion

In summary, although there are several superconducting quantum computing platforms with large qubit number, IBM and Rigetti for instance, we have constructed the most powerful programmable quantum computer to date that has demonstrated algorithms with success rates above the BQP threshold. We have used a trapped ion quantum computer to perform the largest quantum implementations of the BV and HS algorithms. Using a 10-qubit register, we implement all 1024 possible oracles for each algorithm. We exceed the BQP threshold for 87.8% of the oracle implementations in the BV algorithm, an application designed to define this complexity class. Our worst-case oracle implementation, when taking into account detection and preparation error, had a success probability of 50.2%. This implies that it would take <11,500 repetitions to reach the BQP threshold on our worst case oracle. We also demonstrate 35% overlap between the measured and expected output states in the implementation of the HS algorithm, which is a more demanding application due to its higher gate count and exponential speed up over its classical analog. The success of both algorithms is a result of high-fidelity native gates and efficient gate compilation and compression in the fully connected ion trap system. The demonstration of these two canonical algorithms is a starting point for benchmarking any quantum computer. Computing real problems on larger systems with more qubits will require even more gates in the future with even higher quality, and similar standard algorithms to those demonstrated here will likely play a crucial role in benchmarking quantum computers in the future.

## Supplementary information

Supplementary Information

## Data Availability

The data presented in this manuscript are available from the corresponding author upon reasonable request.

## References

[1] Barends R (2014). Superconducting quantum circuits at the surface code threshold for fault tolerance. Nature.

[2] Monz T (2016). Realization of a scalable shor algorithm. Science.

[3] Debnath S (2016). Demonstration of a small programmable quantum computer with atomic qubits. Nature.

[4] Roy, T. et al. A programmable three-qubit superconducting processor with all-to-all connectivity. Preprint at https://arxiv.org/abs/1809.00668 (2018).

[5] Erhard, A. et al. Characterizing large-scale quantum computers via cycle benchmarking. Preprint at https://arxiv.org/abs/1902.08543 (2019). 10.1038/s41467-019-13068-7 PMC6877623 31767840

[6] Chow JM (2012). Universal quantum gate set approaching fault-tolerant thresholds with superconducting qubits. Phys. Rev. Lett..

[7] Devitt SJ (2016). Performing quantum computing experiments in the cloud. Phys. Rev. A.

[8] Pokharel B, Anand N, Fortman B, Lidar DA (2018). Demonstration of fidelity improvement using dynamical decoupling with superconducting qubits. Phys. Rev. Lett..

[9] Hong, S. S. et al. Demonstration of a parametrically-activated entangling gate protected from flux noise. Preprint at https://arxiv.org/abs/1901.08035 (2019).

[10] Nam, Y., et al. Ground-state energy estimation of the water molecule on a trapped ion quantum computer. Preprint at https://arxiv.org/abs/1902.10171 (2019).

[11] Harty TP (2014). High-fidelity preparation, gates, memory, and readout of a trapped-ion quantum bit. Phys. Rev. Lett..

[12] Gaebler JP (2016). High-fidelity universal gate set for $${}^{9}{{\rm{Be}}}^{+}$$ ion qubits. Phys. Rev. Lett..

[13] Ballance CJ, Harty TP, Linke NM, Sepiol MA, Lucas DM (2016). High-fidelity quantum logic gates using trapped-ion hyperfine qubits. Phys. Rev. Lett..

[14] Crain S (2019). High-speed, low-crosstalk detection of a trapped $${}^{171}{{\rm{Yb}}}^{+}$$ ion ancilla qubit using superconducting nanowire single photon detectors. Commun. Phys..

[15] Landsman KA (2019). Verified quantum information scrambling. Nature.

[16] Zhang J (2017). Observation of a many-body dynamical phase transition with a 53-qubit quantum simulator. Nature.

[17] Bohnet JG (2016). Quantum spin dynamics and entanglement generation with hundreds of trapped ions. Science.

[18] Linke NM (2017). Experimental comparison of two quantum computing architectures. Proc. Natl Acad. Sci. USA.

[19] Murali, P. et al. Full-stack, real-system quantum computer studies: technology comparisons and architectural insights. In *ISCA Submission 1300* (2019).

[20] Maunz, P. L. W. *High optical access trap 2.0* https://www.osti.gov/servlets/purl/1237003 (2016).

[21] Fallek SD (2016). Transport implementation of the Bernstein–Vazirani algorithm with ion qubits. New J. Phys..

[22] Hayes D (2010). Entanglement of atomic qubits using an optical frequency comb. Phys. Rev. Lett..

[23] Sørensen A, Mølmer K (1999). Quantum computation with ions in thermal motion. Phys. Rev. Lett..

[24] Choi T (2014). Optimal quantum control of multimode couplings between trapped ion qubits for scalable entanglement. Phys. Rev. Lett..

[25] Wu Y, Wang S-T, Duan L-M (2018). Noise analysis for high-fidelity quantum entangling gates in an anharmonic linear paul trap. Phys. Rev. A.

[26] Grzesiak, N. et al. Efficient arbitrary simultaneously entangling gates on a trapped-ion quantum computer. Preprint at https://arxiv.org/abs/1905.09294 (2019). 10.1038/s41467-020-16790-9 PMC7289877 32528164

[27] Blumel, R., Grzesiak, N. & Nam, Y. Power-optimal, stabilized entangling gate between trapped-ion qubits. Preprint at https://arxiv.org/abs/1905.09292 (2019).

[28] Knill E (2008). Randomized benchmarking of quantum gates. Phys. Rev. A.

[29] Bernstein E, Vazirani U (1997). Quantum complexity theory. SIAM J. Comput..

[30] Grover LK (1997). Quantum computers can search arbitrarily large databases by a single query. Phys. Rev. Lett..

[31] Rötteler, M. Quantum algorithms for highly non-linear Boolean functions. In *Proc. 21st Annual ACM-SIAM Symposium on Discrete Algorithms (SODA’10)* 448–457 (2010).

[32] van Dam W, Hallgren S, Ip L (2006). Quantum algorithms for some hidden shift problems. SIAM J. Comput..

