# Supplementary material for: Benchmarking an 11-qubit quantum computer
Source: Nat Commun. 2019 Nov 29;10:5464. doi: 10.1038/s41467-019-13534-2 (PMC6884641; doi:10.1038/s41467-019-13534-2)
Supplement: Supplementary file 1 — Supplementary Information [file 41467_2019_13534_MOESM1_ESM.pdf]

Benchmarking an 11-qubit quantum computer

Wright, et. al.

November 2019

1 Supplemental Information

The supplemental information presented here is intended to provide additional information to the reader regarding the single- and two-qubit gates used in this manuscript.

Ion	Gate Fidelity	SPAM from RB	SPAM from Microwave
0	99.57(5)	99.31(9)	99.82(4)
1	99.62(6)	99.1(1)	99.77(5)
2	99.18(7)	99.3(1)	99.78(5)
3	99.25(9)	99.6(2)	99.78(5)
4	99.40(9)	99.3(2)	99.84(4)
5	99.46(3)	99.32(7)	99.77(5)
6	99.48(3)	99.27(6)	99.82(4)
7	99.55(4)	99.40(8)	99.83(4)
8	99.59(3)	98.94(6)	99.80(4)
9	99.64(2)	99.35(4)	99.79(5)
10	99.32(6)	99.3(1)	99.79(5)

Table 1: Single-qubit randomized benchmarking (RB) results and microwave SPAM results expressed in percentage (%). To determine single-qubit fidelities for each qubit we apply laser pulses to perform randomized benchmarking for $\pi/2$ gates, using π gates to randomize the computational axes. The data are fit to a power law as described in the text. The average single-qubit fidelity is 99.5%. We can obtain SPAM errors from either the RB results or from a microwave pulse. For microwaves we tune the frequency of the microwave to the qubit splitting and the pulse time is set to drive a spin flip from $|0\rangle \rightarrow |1\rangle$, where the fidelity of detecting the $|1\rangle$ state is the measured SPAM fidelity. The average SPAM fidelity from RB is 99.3% and with microwave based operations the average SPAM fidelity is 99.80%. The uncertainties for the RB results are errors from the fit to a power law (see text) and the uncertainties for microwaves are statistical errors on a binomial distribution, $\sqrt{\frac{P_{|1\rangle}(1-P_{|1\rangle})}{n_{expt}}}$, set by the photon counting statistics.

1	2	3	4	5	6	7	8	9	10	Ion 0 / Ion 1
$98.5^{+0.1}_{-0.3}$	$97.7^{+0.4}_{-0.5}$	$98.5^{+0.1}_{-0.3}$	$97.2^{+0.4}_{-0.5}$	$98.5^{+0.1}_{-0.3}$	$96.9^{+0.5}_{-0.5}$	$97.2^{+0.3}_{-0.5}$	$98.7^{+0.4}_{-0.5}$	$95.5^{+0.4}_{-0.6}$	$97.1^{+0.1}_{-0.3}$	0
	$97.7^{+0.4}_{-0.6}$	$98.9^{+0.1}_{-0.3}$	$98.2^{+0.1}_{-0.3}$	$97.4^{+0.1}_{-0.3}$	$97.8^{+0.1}_{-0.3}$	$98.1^{+0.1}_{-0.3}$	$98.4^{+0.1}_{-0.3}$	$97.7^{+0.3}_{-0.5}$	$97.9^{+0.1}_{-0.3}$	1
		$98.0^{+0.2}_{-0.3}$	$97.5^{+0.3}_{-0.4}$	$96.5^{+0.5}_{-0.6}$	$98.4^{+0.1}_{-0.3}$	$98.0^{+0.1}_{-0.3}$	$97.2^{+0.3}_{-0.5}$	$97.3^{+0.1}_{-0.3}$	$96.0^{+0.6}_{-0.6}$	2
			$96.4^{+0.4}_{-0.5}$	$97.4^{+0.1}_{-0.3}$	$97.1^{+0.4}_{-0.5}$	$98.9^{+0.1}_{-0.3}$	$96.0^{+0.3}_{-0.5}$	$98.0^{+0.1}_{-0.3}$	$97.7^{+0.1}_{-0.3}$	3
				$98.6^{+0.3}_{-0.6}$	$97.3^{+0.4}_{-0.4}$	$97.3^{+0.5}_{-0.5}$	$98.3^{+0.4}_{-0.5}$	$97.8^{+0.1}_{-0.3}$	$96.5^{+0.5}_{-0.6}$	4
					$96.5^{+0.4}_{-0.6}$	$97.1^{+0.3}_{-0.5}$	$98.4^{+0.3}_{-0.4}$	$95.1^{+0.5}_{-0.7}$	$96.7^{+0.5}_{-0.6}$	5
						$96.2^{+0.4}_{-0.6}$	$97.2^{+0.3}_{-0.6}$	$98.1^{+0.4}_{-0.5}$	$98.2^{+0.4}_{-0.5}$	6
							$97.3^{+0.4}_{-0.6}$	$98.5^{+0.3}_{-0.3}$	$97.3^{+0.4}_{-0.6}$	7
								$96.7^{+0.4}_{-0.5}$	$97.0^{+0.3}_{-0.6}$	8
									$97.5^{+0.4}_{-0.5}$	9

Table 2: Raw fidelity of native two-qubit gates expressed in percentage (%). For each qubit pair, we perform the gate and measure the joint populations of pair qubits as a function of analysis pulse phase angle to determine the parity contrast of the created Bell state. The resulting parity and joint-population are determined using maximum likelihood estimation to extract the fidelities enumerated above. The uncertainties are the 1σ confidence interval determined from maximum likelihood estimation. The average fidelity is 97.5% with a minimum and maximum fidelity of 95.1% and 98.9% respectively.
